# Transcriptome Analysis of *Novosphingobium pentaromativorans* US6-1 Reveals the Rsh Regulon and Potential Molecular Mechanisms of *N*-acyl-l-homoserine Lactone Accumulation

**DOI:** 10.3390/ijms19092631

**Published:** 2018-09-05

**Authors:** Hang Lu, Yili Huang

**Affiliations:** Zhejiang Provincial Key Laboratory of Organic Pollution Process and Control, Department of Environmental Science, College of Environmental and Resource Sciences, Zhejiang University, Hangzhou 310027, China; 21514007@zju.edu.cn

**Keywords:** Rsh regulon, *Novosphingobium pentaromativorans* US6-1, sphingomonads, RNA-seq, *N*-acyl-l-homoserine lactone, ppGpp

## Abstract

In most bacteria, a bifunctional Rsh responsible for (p)ppGpp metabolism is the key player in stringent response. To date, no transcriptome-wide study has been conducted to investigate the Rsh regulon, and the molecular mechanism of how Rsh affects the accumulation of *N*-acyl-l-homoserine lactone (AHL) remains unknown in sphingomonads. In this study, we identified an *rsh*_US6–1_ gene by sequence analysis in *Novosphingobium pentaromativorans* US6-1, a member of the sphingomonads. RNA-seq was used to determine transcription profiles of the wild type and the ppGpp-deficient *rsh*_US6–1_ deletion mutant (∆*rsh*). There were 1540 genes in the Rsh_US6–1_ regulon, including those involved in common traits of sphingomonads such as exopolysaccharide biosynthesis. Furthermore, both RNA-seq and quantitative real-time polymerase chain reaction (qRT-PCR) showed essential genes for AHL production (*novI* and *novR*) were positively regulated by Rsh_US6–1_ during the exponential growth phase. A degradation experiment indicated the reason for the AHL absence in ∆*rsh* was unrelated to the AHL degradation. According to RNA-seq, we proposed σ^E^, DksA, Lon protease and RNA degradation enzymes might be involved in the Rsh_US6–1_-dependent expression of *novI* and *novR*. Here, we report the first transcriptome-wide analysis of the Rsh regulon in sphingomonads and investigate the potential mechanisms regulating AHL accumulation, which is an important step towards understanding the regulatory system of stringent response in sphingomonads.

## 1. Introduction

Bacteria need to co-ordinate cellular responses to unfavorable environmental conditions [1]. One major strategy to cope with environmental stress is the activation of stringent response, a global regulatory system [2]. The stringent response is activated by (p)ppGpp (guanosine tetraphosphate and guanosine pentaphosphate) [3]. The proteins from the RelA/SpoT (Rsh) family are the key players, synthesizing (p)ppGpp from ATP and either GTP or GDP, and degrading (p)ppGpp to pyrophosphate and either GTP or GDP. Most species in *Betaproteobacteria* and *Gammaproteobacteria* contain two multi-domain Rsh enzymes, RelA and SpoT, while the majority of bacteria contain only a single Rsh protein [3]. A number of studies have demonstrated that Rsh affected the expression of a wide range of genes involved in physiological processes in bacteria such as *Escherichia coli*, *Staphylococcus aureus* and *Rhizobium etli* [4,5,6]. In *R. etli*, there were 834 genes in the Rsh regulon involved in various cellular processes such as transcriptional regulation, signal transduction, production of sigma factors and non-coding RNAs [6]. Sphingomonads, a group of *Alphaproteobacteria*, are widely distributed in polluted and oligotrophic environments [7,8]. Sphingomonads have drawn much attention for their traits, including the pronounced abilities of degrading a wide range of recalcitrant natural and xenobiotic compounds such as polycyclic aromatic hydrocarbons (PAH) [7,9], the substitution of sphingolipids for lipopolysaccharide in their outer membrane [10,11] as well as their production of exopolysaccharides (EPS) [12,13]. The genomes of sphingomonads contain one single *rsh* gene, which responds to environmental stress as in other bacteria [14]. However, to date, no transcriptome-wide study has been conducted to investigate Rsh target genes in any strain of sphingomonads.

One of the physiological activities regulated by Rsh is quorum sensing (QS), a mechanism of intercellular communication. In Gram-negative bacteria, the main QS signal is *N*-acyl-l-homoserine lactone (AHL), which is produced by LuxI-type synthases. LuxR-type receptors can bind to AHL and then stimulate the expression of *luxI* homologs [15]. AHL accumulation is dependent on RelA/SpoT homologs in various bacteria such as *Pseudomonas aeruginosa* [16,17,18] and *R. etli* [19], and AHL degradation is regulated by (p)ppGpp via AttM in *Agrobacterium tumefaciens* [20,21]. In *P. aeruginosa*, deletion of both *relA* and *spoT* resulted in increased levels of 4-hydroxyl-2-heptylquinoline and 3,4-dihydroxy-2-heptylquinoline via up-regulated *pqsA* and *pqsR* expression and decreased levels of butanoyl-homoserine lactone and 3-oxo-dodecanoyl-homoserine lactone via down-regulated *rhlI*, *rhlR*, *lasI*, and *lasR* expression [18]. In recent years, an increasing number of strains in sphingomonads which produced AHLs have been isolated [22]. The comparative genomic analyses of 62 sphingomonads genomes showed that the canonical *luxI/R*-type QS network was widespread within sphingomonads [23]. In the previous study, a Tn*5* mutant of *Novosphingobium* sp. Rr 2-17, deficient in AHL accumulation, was found to have an insertion in an *rsh* gene, suggesting that QS was under the regulation of Rsh in *Novosphingobium*, a member of sphingomonads. However, the potential molecular mechanism remains unknown.

*Novosphingobium pentaromativorans* US6-1, which has been shown to have potential in aromatic hydrocarbons bioremediation, is a type strain belonging to sphingomonads [24]. Its genome sequencing has been completed and the genome database is accessible from the public NCBI database [25]. In this study, we identified an *rsh* gene in *N. pentaromativorans* US6-1 (annotated as *rsh*_US6–1_) by sequence analysis. The wild-type strain produced ppGpp in static culture medium while *rsh*_US6–1_ deletion mutant (∆*rsh*) did not. Therefore, the transcription profiles of the wild type and ∆*rsh* grown in static medium was determined by RNA-seq to identify the Rsh_US6–1_ regulon. Furthermore, we determined whether AHL accumulation was affected by Rsh_US6–1_ and investigated the potential molecular mechanisms via quantitative real-time polymerase chain reaction (qRT-PCR), transcriptome analysis and an AHL degradation experiment. These results are useful to understand the regulatory system of stringent response in sphingomonads.

## 2. Results and Discussion

### 2.1. Sequence Analysis of Rsh_US6–1_ Protein

**Figure 1:**
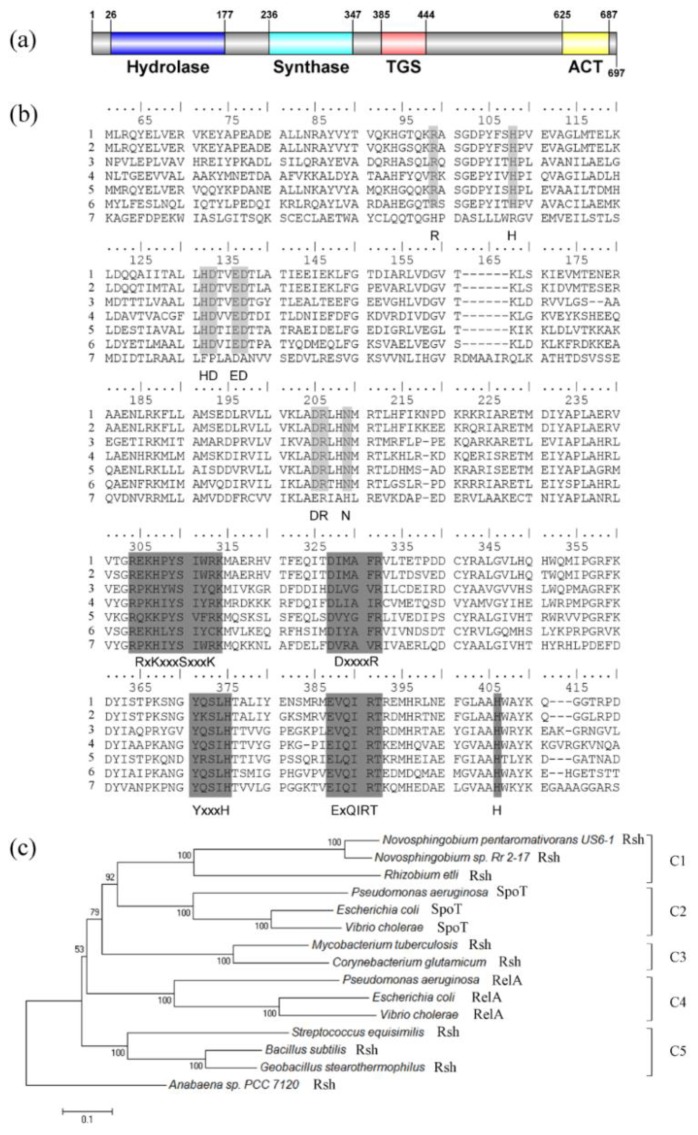
Sequence analysis of Rsh_US6–1_ protein. (**a**) Rsh_US6–1_ domain; (**b**) amino acid alignment of RelA/SpoT homologs. Line 1: Rsh_US6–1_. Line 2: Rsh_Rr2–17_. Line 3: Rsh_Mtb_. Line 4: Rsh_Seq_. Line 5: Rsh_Ret_. Line 6: SpoT_Ecoli_. Line 7: RelA_Ecoli_ (see in Materials and Methods). Light grey boxes represent six conserved motifs in HD domain (HD 1: R. HD 2: H. HD 3: HD. HD 4: ED. HD 5: DR. HD 6: N) while dark grey boxes represent five conserved motifs in Syn domain (Syn 1: RxKxxxSxxxK. Syn 2: DxxxxR. Syn 3: YxxxH. Syn 4: ExQIRT. Syn 5: H) [26]; (**c**) phylogenetic tree based on RelA/SpoT homologs. Sequences, available at NCBI GenBank, were aligned by using the ClustalW algorithm [27] in MEGA version 6.0 [28]. Then a phylogenetic tree was constructed by the neighbor-joining method [29] with 1000 bootstrap replicates [30] after cutting off the redundant sequences at the end, with the use of MEGA version 6.0. The Rsh protein from *Anabaena sp.* PCC 7120 (accession number BAB77915) was used as outgroup [31]. There were five clusters: C1 for Rsh in *Alphaproteobacteria*, C2 for SpoT in *Gammaproteobacteria*, C3 for Rsh in *Actinobacteria*, C4 for RelA in *Gammaproteobacteria*, C5 for Rsh in *Firmicutes*.

The open reading frame of the full-length *rsh*_US6–1_ gene (accession number WP_007011921) was 2094 nucleotides in length. Rsh_US6–1_ contained the nitrogen-terminal metal-dependent hydrolase domain (HD) (amino acids 26–177) and synthetase domain (Syn) (amino acids 236–347). The carboxy-terminal domain of Rsh_US6–1_ consisted of the TGS domain (for: Thr-tRNA synthetase, GTPase and SpoT) (amino acids 385–444) and ACT domain (for: aspartate kinase, chorismate mutase and T protein) (amino acids 625–687) (Figure 1a). These four domains are commonly present in Rsh [3].

Rsh_US6–1_ displayed high sequence similarity to Rsh proteins whose functions had been identified in other strains [32,33,34,35,36]. The identities of Rsh_US6–1_ with Rsh_Rr2–17_, Rsh_Mtb_, Rsh_Seq_, Rsh_Ret_, SpoT_Ecoli_ and RelA_Ecoli_ were 88%, 37%, 38%, 43%, 38% and 30% respectively. In the HD domain (Figure 1b), Rsh_US6–1_ contained six motifs, which are predicted to be essential for (p)ppGpp hydrolysis [26]. RelA_Ecoli_ was more divergent in these motifs, which was consistent with its loss of hydrolase activity. In the Syn domain (Figure 1b), Rsh_US6–1_ also contained five conserved motifs that were proved structurally and biochemically important for (p)ppGpp synthetase activity [26].

The phylogenic analysis of RelA/SpoT homologs was performed (Figure 1c). The result showed that there was a clear separation of the RelA/SpoT families, forming five clusters: C1 for Rsh in *Alphaproteobacteria*, C2 for SpoT in *Gammaproteobacteria*, C3 for Rsh in *Actinobacteria*, C4 for RelA in *Gammaproteobacteria*, C5 for Rsh in *Firmicutes*. Rsh_US6–1_ was grouped with Rsh_Rr2–17_ in C1. These data suggested that Rsh_US6–1_ possessed the sequence required for the (p)ppGpp synthetase and hydrolase activities.

### 2.2. N-Acyl-l-homoserine Lactone (AHL) Accumulation in the Cross Feeding Assay and Extract Assay

**Figure 2:**
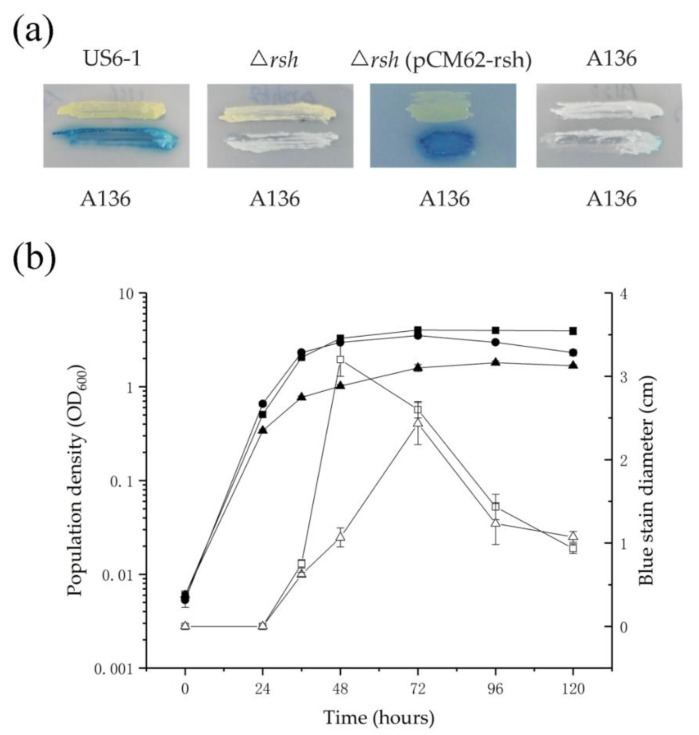
AHL accumulation in US6-1, ∆*rsh and* ∆*rsh* (pCM62-rsh). (**a**) AHL accumulation assay by cross-feeding. P5Y3 agar plate was covered with 50 µL X-gal. The AHL reporter strain A136 and the tested strains were streaked side by side on the agar plates. Plates were incubated for 24 h, when the activation of the reporter was recorded. Strain A136 versus A136 was used as negative control. One representative experiment out of three independent biological replicates is shown. (**b**) Time course of population density of US6-1 (filled squares), ∆*rsh* (filled circles) and ∆*rsh* (pCM62-rsh) (filled triangles) and AHL accumulation of US6-1 (open squares) and ∆*rsh* (pCM62-rsh) (open triangles) in the extract assay. Fresh colonies of strains were first inoculated into P5Y3 broth at the shaking speed of 200 rpm to an OD_600_ value of 1. Then 250 µL of this seed culture was re-inoculated into 50 mL of fresh P5Y3 broth and incubated statically. The growth curve was drawn from measuring the OD_600_ values. AHL signals in cultures were then extracted by ethyl acetate (EA). The extracts were spotted onto LB soft agar plates plus X-gal mixed with A136. The plates were incubated for 12 h at 30 °C and the diameters of blue stains which represented the quantity of AHL signals in bacterial cultures were measured. No AHL was detected in ∆*rsh*. Values shown are the average of biological triplicate experiments with standard deviations marked with error bars.

To determine the effect of Rsh_US6–1_ on the AHL accumulation, cross-feeding and extract assays were performed. The results of the cross-feeding and extract assays were consistent (Figure 2). In the cross-feeding assay, strains were grown as biofilm and only US6-1 showed AHL accumulation. In the extract assay, US6-1 accumulated AHL signals in static cultures while no AHL was detected in ∆*rsh* during the whole growth period. To verify that the *rsh*_US6–1_ deletion was responsible for the absence of AHL, a *rsh*_US6–1_ complementation strain ∆*rsh* (pCM62-rsh) was created. ∆*rsh* (pCM62-rsh) restored the same phenotype as the wild-type strain, suggesting that Rsh_US6–1_ was required for the AHL accumulation. In the extract assay, the AHL molecules in the extract of culture would diffuse through the soft agar to the reporter strain *A. tumefaciens* A136, and activate the *traI*-*lacZ* fusion. Therefore, we could determine the quantity of AHL molecules by the diffusion area indicated by the diameter of the blue coloration in the presence of 5-bromo-4-chloro-3-indolyl-β-d-galactopyranoside (X-Gal) [37]. The relationship between the growth of US6-1 and AHL accumulation was analyzed carefully. The bacteria cells grew exponentially for about 48 h and then entered the stationary phase. Similarly, the AHL accumulation was maximized at 48 h and then declined, indicating US6-1 could degrade AHL signals.

### 2.3. ppGpp Accumulation in Strain US6-1 and Its Derivatives

**Figure 3:**
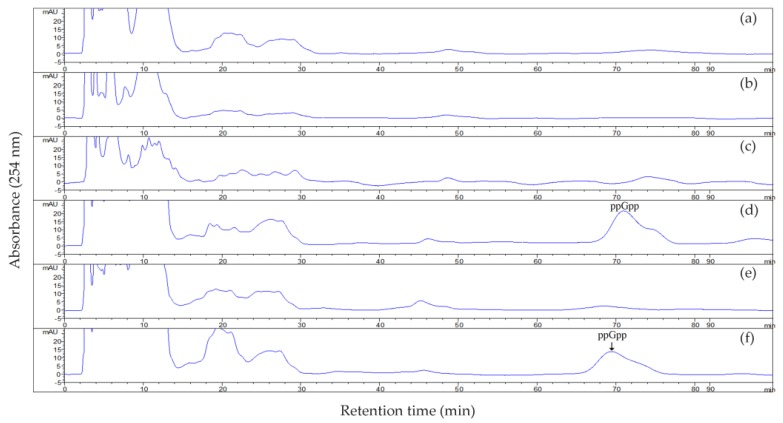
ppGpp accumulation in strain US6-1, ∆*rsh* and ∆*rsh* (pCM62-rsh) in different growth phases. Levels of ppGpp in cultures of US6-1 (**a**,**d**), ∆*rsh* (**b**,**e**) and ∆*rsh* (pCM62-rsh) (**c**,**f**) in the exponential growth phase (36 h) and in the stationary phase (72 h) were monitored by reverse phase high-performance liquid chromatography (HPLC) analysis. The eluted nucleotides were monitored at 254 nm and identified by comparison with the retention time of 100 µM ppGpp standard. ppGpp standard was eluted at 71 min under the current conditions and the position is indicated by an arrow.

We monitored the endogenous ppGpp levels in US6-1, ∆*rsh* and ∆*rsh* (pCM62-rsh) (Figure 3). The presence of ppGpp in US6-1 culture at 72 h of incubation (stationary phase) was determined while no ppGpp was detected in ∆*rsh.* When we analyzed ppGpp accumulation in ∆*rsh* (pCM62-rsh), the complementation of ppGpp production was observed, confirming that Rsh_US6–1_ was responsible for ppGpp synthesis. These results showed that the stringent response in US6-1 and the complementary strain was induced under the current culture conditions. Nutrient limitation is one of the conditions that can induce the stringent response. However, the medium used in this study was nutrient-rich P5Y3 medium. Several studies has recently found that antibiotics, acid stress and oxidative stress could also induce the stringent response [38]. Therefore, it seems that many factors which can induce the stringent response of bacteria remain unknown. At 36 h of incubation (exponential growth phase), we could not detect the ppGpp accumulation in US6-1 culture. This was probably because the ppGpp level was below the detectable range, as ppGpp is generally thought to be present at basal levels in the exponential growth phase [3]. However, previous studies showed that ppGpp could still regulate the expression of a wide range of genes during this growth period [3,6].

### 2.4. Global Overview of the Rsh_US6–1_ Regulon

The RNA-seq of strain US6-1 and ∆*rsh* cultivated in static conditions, in which US6-1 accumulated AHL signals, was conducted to determine the Rsh_US6–1_ regulon. An average of 24 million clean reads in one sample were obtained. The total mapping radios and uniquely mapping ratios of clean reads to reference genome of US6-1 were up to 98% and 95%, indicating that the sequencing was deep enough to cover almost all kinds of transcripts in the cells (Table S1). The genome of US6-1 contains 5110 annotated protein-encoding genes, 59 pseudo genes and 82 RNA genes [25]. The comparative transcriptome analysis between strain US6-1 and ∆*rsh* revealed 1540 Rsh_US6–1_-dependent differentially expressed genes (DEGs), which were defined according to the combination of the absolute value of a fold change ≥2 and an adjust *p* value ≤ 0.05 (Table S2). Compared with the wild type, 911 of these genes were up-regulated (17.83%) and 629 genes (12.31%) were down-regulated in ∆*rsh* (*∆rsh* vs. US6-1). The differential expression varied between an 8.82-fold up-regulation of a gene encoding a protein of ferrisiderophore receptor (WP_007014902) and a 40.5-fold down-regulation of an autoinducer synthesis protein (WP_007013362). The transcriptome data were validated by analyzing the expression levels of 39 representative genes using qRT-PCR (Table S3). The result showed that 29 (74.36%) of the tested genes were regulated in the same direction (up or down) in both RNA-seq data and qRT-PCR (∆*rsh* vs. US6-1), and the fold change of each gene in qRT-PCR was log_2_(x) > 1 or log_2_(1/x) < −1 (representing a plain fold-change >2) with statistical differences. Among these 29 genes, the expression of 24 genes by qRT-PCR (the complementary strain vs. US6-1) was not different significantly or was regulated in the opposite direction in the RNA-seq data. Therefore, the transcriptome data were in good agreement with the qRT-PCR data, which proved the reliability of the transcriptome data.

**Figure 4:**
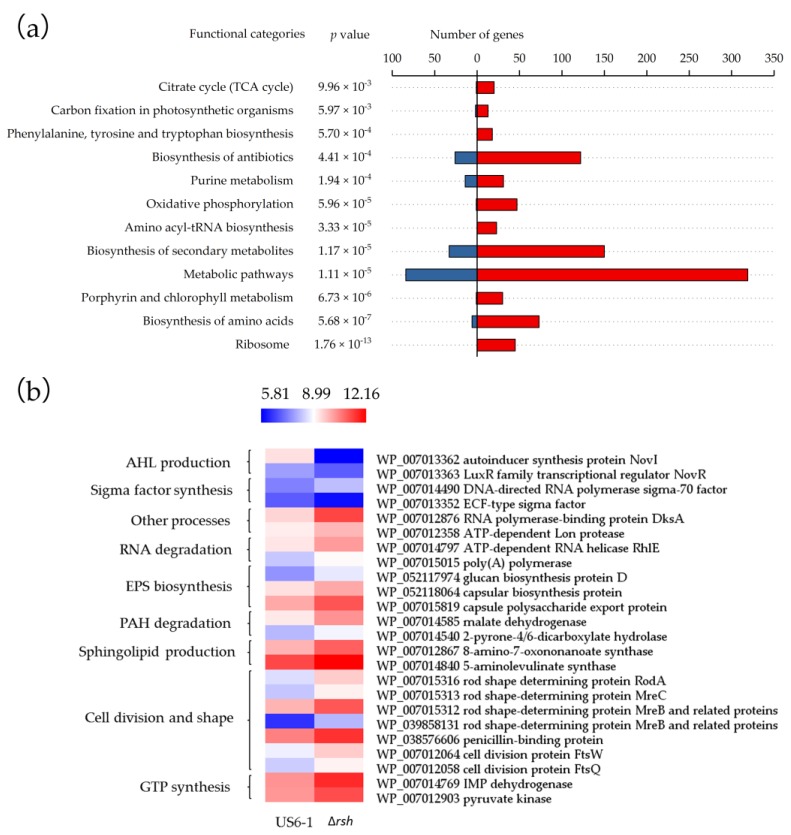
Differentially expressed genes (DEGs) overview (∆*rsh* vs. US6-1) in the RNA-seq data. (**a**) Over-represented Kyoto Encyclopedia of Genes and Genomes (KEGG) categories. KEGG annotation and pathway enrichment analysis was performed using the KEGG pathway database (http://www.genome.jp/kegg/). The *p* values denote the enriched levels in a KEGG pathway, which was calculated using a Fisher’s exact test [42]. Up- and down-regulated genes (∆*rsh* vs. US6-1) are represented by red and blue bars respectively, representing the number of genes per functional category. (**b**) Heat map of log_2_ expression ratios of specific DEGs involved in AHL production, sigma factor synthesis, RNA degradation, EPS biosynthesis, PAH degradation, sphingolipid production, cell division and shape, GTP synthesis and other functions in strain US6-1 and ∆*rsh*. Expression values are reflected by red-blue coloring as indicated. The heat map was drawn by the software HemI [43].

These genes were further grouped based on Kyoto Encyclopedia of Genes and Genomes (KEGG) annotation and pathway enrichment analysis (Figure 4a). The Fisher’s exact test identified 12 significantly over-represented KEGG pathways. The result suggested that Rsh_US6–1_ controlled a variety of metabolic pathways. The most of the DEGs involved in ribosomal protein production, porphyrin and chlorophyll metabolism, amino acyl-tRNA biosynthesis, oxidative phosphorylation, TCA cycle, phenylalanine, tyrosine and tryptophan biosynthesis were up-regulated in the mutant, which suggested that the role of Rsh_US6–1_, similar to that in *E. coli*, was to repress the majority of cellular processes to reallocate cellular resources [39]. Although one of the hallmarks of stringent response is the induction of amino acid biosynthesis [39,40], unexpectedly, in ∆*rsh* most genes involved in amino acid biosynthesis were up-regulated. Similarly, in *relA* mutant of *Pectobacterium atrosepticum*, several unlinked clusters of genes involved in branched chain amino acid metabolism (*ilvGMEDA*, *ilvlH*, *ilvBN* and *leuABCD*) were also up-regulated [41]. There may be other mechanisms which can induce amino acid biosynthesis when US6-1 grows under environmental stress. According to the validation of the transcriptome data, DEGs involved in AHL production, sigma factor synthesis, RNA degradation, EPS biosynthesis, PAH degradation, sphingolipid production, cell division and shape and GTP synthesis were chosen, and their expression profiles were visualized in the heat map (Figure 4b, Table S3). Except the genes related to AHL production, the most of these genes were under negative control of Rsh_US6–1_. These DEGs will be discussed in detail in the following paragraphs.

### 2.5. Essential Genes for AHL Production were Positively Regulated by Rsh_US6–1_ in the Exponential Growth Phase

There are only one gene *novI*, encoding an autoinducer synthase (WP_007013362) and one gene *novR* encoding a LuxR family transcriptional regulator containing an autoinducer binding domain (WP_007013363) in the genome of US6-1. These two genes are essential for AHL production. The transcriptome data showed that in strain ∆*rsh*, *novI* and *novR* were down-regulated 40.5 and 2.35-fold at 36 h of incubation, respectively. The qRT-PCR analysis of culture samples from different growth periods confirmed the decreased expression levels of *novI* and *novR* in ∆*rsh* (Table 1). The *novI* and *novR* were both down-regulated significantly at 36 h and 48 h (exponential growth phase) in ∆*rsh* while ∆*rsh* (pCM62-rsh) could complemented their expression.

**Figure 5:**
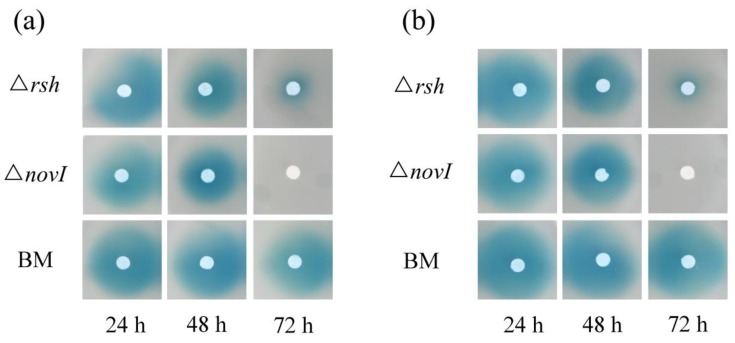
Identification of degradation of the medium-chain AHL by ∆*rsh*. Strain ∆*rsh* was incubated with 2 µM C8-AHL (**a**) and 3-OH-C8-AHL (**b**) in culture medium for 24 h (early exponential growth phase), 48 h (late exponential growth phase) and 72 h (stationary phase), after which the EA extracts were spotted onto AHL reporter plates mixed with A136. The diameters of the blue stains represent the quantity of residual AHL signals. To avoid the influence of the AHL produced by strain US6-1 in the exponential growth phase, *novI* deletion mutant (∆*novI*) which could not produce AHL was used as positive control. Blank medium (BM) with AHL was used as negative control.

Although the essential genes for AHL production were down-regulated in the exponential growth phase due to the lack of ppGpp, ∆*rsh* still possibly produced low concentrations of AHL signals. Since US6-1 could degrade AHL signals, we analyzed whether ∆*rsh* degraded AHL in the exponential growth phase. When provided with 2 µM medium-chain AHL (C8-AHL and 3-OH-C8-AHL) as cosubstrates in culture medium, ∆*rsh* did not show the capacity to degrade the medium-chain AHL at 48 h (Figure 5). However, at 72 h of incubation, there were fewer AHL signals in ∆*rsh* cultures. These results suggested that ∆*rsh* might not degrade AHL signals in the exponential growth phase. The activation of its capacity to degrade AHL was likely related to the entrance to the stationary phase. Overall, the reason why Rsh_US6–1_ was required for the AHL accumulation was that Rsh_US6–1_ positively regulated essential genes for AHL production in the exponential growth phase, and might be unrelated to the AHL degradation.

### 2.6. Potential Molecular Mechanisms of Rsh_US6–1_-Dependent Expression of novI and novR

In the case of *E. coli*, (p)ppGpp regulates the expression of genes through binding to β’-subunit of RNA polymerase to destabilize the short-lived open complexes that form at certain promoters [3]. However, genes in the Rsh_US6–1_ regulon were numerous and the proteins which can modulate the expression of *luxI/R* homologs vary in bacteria [44]. Therefore, the transcriptome data were used to investigate the potential molecular mechanisms of the decreased expression of *novI* and *novR* in *∆rsh*.

During exponential growth, transcription is widely under control of the housekeeping sigma factor σ^70^. ppGpp can regulate the expression of genes via the regulation of alternative sigma factor competition [45]. Previous studies showed that QS was influenced by alternative sigma factors. In *P. aeruginosa* PAO1 and *P. fluorescens* UK4, ppGpp up-regulates the expression of *rpo*S encoding a stationary phase sigma factor and RpoS increases the expression level of AHL-related genes as well as the AHL production [16,46]. However, the *Pseudomonas* model might not be applicable in strain US6-1, since its genome does not encode a corresponding RpoS. US6-1 possesses 22 putative sigma factors. Only one housekeeping sigma factor σ^70^ (WP_007014490) was up-regulated 2.2-fold while a extracytoplasmic function (ECF) sigma factor (WP_007013352) was down-regulated 2.73-fold in ∆*rsh*. The link between ECF sigma factor and QS has recently given rise to controversy. ECF sigma factors can bind directly to -10 and -35 elements in the *luxR* promoter, thus inducing *luxR* expression. The binding sites of this kind of sigma factors are also identified in the upstream region of *luxS* [47,48]. However, the effect of ECF sigma factors on QS was not observed in another study [49].

Our data showed that *dksA* (WP_007012876) was up-regulated significantly (6.41-fold) in ∆*rsh*. DksA, a (p)ppGpp cofactor, can bind the secondary channel and sensitize RNAP to (p)ppGpp at many promoters [3]. The previous study showed that DksA could also function without ppGpp [50]. In *P. aeruginosa*, DksA represses the expression of *rhlI* during the exponential growth [51].

The gene encoding an ATP-dependent Lon protease (WP_007012358) was up-regulated 2.01-fold due to the Rsh_US6–1_ absence. The gene is involved in the toxin–antitoxin (TA) module, a (p)ppGpp-dependent mechanism related to antibiotic tolerance and resistance [3]. Lon protease is a powerful negative regulator of two HSL-mediated QS systems in *P. aeruginosa* [52]. In the TA model, (p)ppGpp activates the Lon protease [3]. Interestingly, our data indicated an opposite type of the TA module regulation of ppGpp in strain US6-1.

Moreover, the expression levels of two genes involved in the putative functions of RNA degradation (WP_007014797 and WP_007015015) were increased 2.62 and 2.19-fold respectively in ∆*rsh*. The enzyme responsible for RNA degradation has been reported to degrade mRNA of AHL synthase genes and thus decrease the AHL production rapidly [53]. Overall, the above transcriptome data showed that ppGpp could regulate several factors including the ECF sigma factor, DksA, ATP-dependent Lon protease and RNA degradation enzymes. These factors have been proved to affect the expression of *luxI/R* homologs in other bacteria. However, the relationship between these factors and *luxI/R* homologs in sphingomonads remains unknown and needs further studies. We proposed these potential molecular mechanisms of ppGpp-dependent expression of *novI* and *novR*, which might contribute to giving insight into the complicated cross-talk between stringent response and QS in sphingomonads.

### 2.7. Repressed Exopolysaccharides (EPS) Biosynthesis

**Figure 6:**
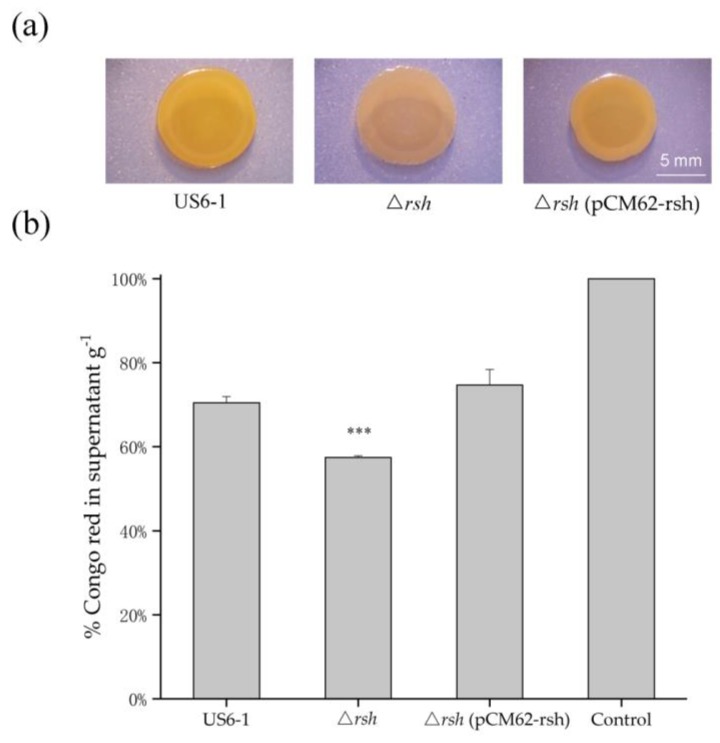
Assessment of EPS production of strain US6-1, ∆*rsh* and ∆*rsh* (pCM62-rsh). (**a**) Colony biofilm morphology of strains on the P5Y3 plates containing 40 μg/mL Congo red. One representative experiment out of three independent biological replicates is shown. The white bar represents 5 mm. (**b**) Congo red binding assay. The biomass of strains grown in static culture for 96 h was determined gravimetrically as cell wet weigh. The bacterial mass was then mixed with 1 mL of Congo red (100 μg/mL) in 0.9% saline for binding. After removing the biomass, the OD_490_ of unbound Congo red in supernatant was measured and the percentage of Congo red left in supernatant g^−1^ was shown. The OD_490_ of 100 μg/mL Congo red in 0.9% saline was used as the control. The results are averages of three replicates, and the error bars indicate standard deviations. “***” represents *p* < 0.001.

Many strains in sphingomonads can produce EPS [12,13]. The transcriptome data showed the gene encoding a putative glucan biosynthesis protein (WP_052117974) and two genes involved in capsule biosynthesis and export (WP_052118064, WP_007015819) were up-regulated 3.03, 2.07 and 3.01-fold respectively in strain ∆*rsh*. Glucans are major constituents of capsular materials [54]. Thus, we analyzed ∆*rsh* for Congo red binding. Congo red binds certain polysaccharides as well as polymers that display amyloid-like properties [12]. On Congo red plates, the colony biofilm of ∆*rsh* was light red, while the wild-type strain appeared slightly orange. The complemented strain showed a phenotype similar to that of the wild-type strain (Figure 6a). Furthermore, the culture of ∆*rsh* bound more Congo red than the wild type, leaving less Congo red in the cell-free supernatant. The Congo red bounding of ∆*rsh* (pCM62-rsh) showed no significant difference with that of strain US6-1 (Figure 6b). The results suggested that Rsh_US6–1_ affected EPS biosynthesis as a repressor, consistent with the changes of the expression levels of the above genes. This phenomenon was also observed in *Sinorhizobium meliloti* [55]. When the local environment becomes unfavorable (e.g., nutrient or oxygen deficiency), the motile behavior is important for bacteria to move towards a better environment to survive. In several strains of sphingomonads, the presence of EPS can result in a phenotypic shift from planktonic cells to non-motile cells [13]. Therefore, the repression of Rsh_US6–1_ on the EPS biosynthesis might be a survival strategy of strain US6-1.

### 2.8. Rsh_US6–1_ Regulon Involved in Other Processes

The large plasmids present in US6-1 possess putative biodegradation genes that play a key role in PAH degradation [56]. These genes were investigated in the transcriptome data. Two genes (WP_007014585, WP_007014540) located in the large plasmid pLA3 showed up-regulation in ∆*rsh*, which was similar to the situation in *Sphingomonas* sp. LH128 [14]. For strain LH128, the expression of phenanthrene catabolic genes were decreased while *rsh* was up-regulated after 6 months of starvation. This indicated that Rsh_US6–1_ might affect the degradation of PAH. Many researches on sphingomonads mainly focused on enzymes which could degrade PAH directly [56]. Considering sphingomonads always degrade aromatic compounds in contaminated soil with limited nutrient availability [7], studying the relationship between Rsh and genes involved in the degradation of aromatic compounds would help understand a comprehensive degradation mechanism. Future studies carried out in an environment contaminated with PAH using ∆*rsh* are needed to investigate the relationship.

All sphingomonads contain in their outer membranes sphingolipids which replace lipopolysaccharides [7]. Serine palmitoyltransferase (SPT) is a key enzyme in sphingolipid biosynthesis [57]. Two up-regulated genes (WP_007012867 and WP_007014840) were annotated as α-oxoamine synthases, a family of enzymes including SPT. Their identification of amino acid sequences with SPT from *Sphingomonas paucimobilis* and *Sphingomonas wittichii* were more than 30% [57,58], indicating they were responsible for encoding SPT. Sphingolipids play an important role in bacterial survival under stress [59]. However, our results indicated that when spingomonads encountered environmental stress, sphingolipid biosynthesis was repressed via stringent response instead of being induced, probably for energy and resource saving.

The expression level of shape determination genes (*rodA*, *mreC* and *mreB*), cell-division genes (*ftsW*, *ftsQ*) and genes encoding penicillin-binding proteins increased as a result of the deletion of *rsh*_US6–1_. These genes were involved in cell size and shape, indicating that the morphology of US6-1 was regulated by Rsh_US6–1_. Starvation stress can result in the changes of cell morphology of *Sphingomonas* [14,60]. The reason might be that the change of cell size and shape improves the uptake of scarce nutrients due to the increase in cell surface area to volume ratio [61]. Our data suggested that US6-1 might adapt to environment stress via the change in cell size or shape regulated by Rsh_US6–1_.

Genes responsible for putative regulatory functions were investigated. Several transcriptional regulators including the cold shock protein, an UspA and a PadR family transcriptional regulator were down-regulated. The balance of available GTP might be altered due to the up-regulation of two genes involved in GTP synthesis, a gene encoding an IMP dehydrogenase (WP_007014769) and a pyruvate kinase (WP_007012903). In *Bacillus subtilis*, Rsh regulates transcription mainly by altering the balance of GTP [3]. Whether Rsh also affects the expression of genes through the changes of GTP concentration in sphingomonads remains unknown. In addition, the genome of US6-1 contains 16 chemotaxis-related genes in a “che” cluster, in which several genes were up-regulated. Many genes involved in iron homeostasis were also in the Rsh_US6–1_ regulon. There was an up-regulation in the expression level of genes encoding transporters ExbD and a ferrous iron transporter B and a 3.48-fold down-regulation in the gene related to bacterioferritin. Among proteins annotated as TonB-dependent receptors, 10 were up-regulated and 13 were down-regulated. The absence of Rsh_US6–1_ seemed to result in a disruption in iron homeostasis.

## 3. Materials and Methods

### 3.1. Bacterial Strains, Plasmids and Growth Conditions

Bacterial strains and plasmids used in this study are listed in Table 2. Strain US6-1 and its derivatives were grown in P5Y3 (5 g/L peptone, 3 g/L yeast exact and 25 g/L sea salt, pH = 6.5 ± 0.2) at 30 °C. Unless noticed otherwise, in all experiments fresh colonies of US6-1 and its derivatives were first inoculated into P5Y3 broth at the shaking speed of 200 rpm to an OD_600_ value of 1 and 250 µL of this seed culture was then re-inoculated into 50 mL of fresh P5Y3 broth and incubated statically. *E. coli* and *A. tumefaciens* A136 were grown in Luria Broth (10 g/L tryptone, 5 g/L yeast extract, 10 g/L NaCl) at 37 °C or 30 °C, respectively. When appropriate, the following antibiotics were added to the medium: kanamycin (100 µg/mL), streptomycin (100 µg/mL), rifampicin (50 µg/mL) and tetracycline (9 µg/mL) for strain US6-1 and its derived strains, kanamycin (50 µg/mL), tetracycline (9 µg/mL) and ampicillin (100 µg/mL) for *E. coli*, tetracycline (4.5 µg/mL) and spectinomycin (50 µg/mL) for A136.

### 3.2. Sequence Analysis of Rsh_US6–1_

The nucleotide sequence of *rsh*_US6–1_ (accession number WP_007011921) and its deduced amino acid sequences were obtained from NCBI GenBank (https://www.ncbi.nlm.nih.gov, 15 November 2016). Domain prediction was carried out using Conserved Domain Database in NCBI. Multiple-sequence alignments were accomplished using BIOEDIT version 7.0.9.0 (Ibis Therapeutics, Carlsbad, CA, USA) [66]. The identities of Rsh_US6-1_ with Rsh_Rr2-17_ (Rsh from *Novosphingobium* sp. Rr 2-17, accession number ACH57394) [33], Rsh_Mtb_ (Rsh from *Mycobacterium tuberculosis*, accession number CAB01260) [34], Rsh_Seq_ (Rsh from *Streptococcus equisimilis*, accession number CAA51353) [35], Rsh_Ret_ (Rsh from *Rhizobium etli*, accession number ABC90188) [36], SpoT_Ecoli_ (SpoT from *E. coli*, accession number AAC76674) [32] and ${\mathrm{RelA}}_{\mathrm{Ecoli}}$ (RelA from *E. coli*, accession number AAC75826) were calculated by BLAST+ program [67]. For construction of phylogenetic trees, sequences were aligned by using the ClustalW algorithm [27] in MEGA version 6.0 [28]. Then a phylogenetic tree was constructed by the neighbor-joining method [29] with 1000 bootstrap replicates [30] after cutting off the redundant sequences at the end, with the use of MEGA version 6.0. The Rsh protein from *Anabaena sp.* PCC 7120 (accession number BAB77915) was used as outgroup [31].

### 3.3. Strain Construction

Primers used in the present study are listed in Table S4. The in-frame gene deletion mutant was constructed as described previously [64]. Briefly, up- and down-stream flanking regions of *rsh*_US6–1_, approximately 400 bp each, were amplified by PCR with the primer pairs rsh-5O/rsh-5I and rsh-3O/rsh-3I and joined using overlap PCR with rsh-5O/rsh-3O. The resulting fragments were cloned into the plasmid pAK405 via the *Bam*HI/*Hind*III restriction site. The pAK405 derivative was subsequently delivered into US6-1 via conjugal transfer from *E. coli* S17-1 (λpir). After the conjugal transfer, bacteria were plated on P5Y3 supplemented with kanamycin (100 μg/mL) and rifampicin (50 µg/mL). Individual colonies were restreaked once on the same medium and then plated on P5Y3 supplemented with 100 μg/mL streptomycin to select for the second homologous recombination event. The resulting colonies were restreaked on both P5Y3 supplemented with 100 µg/mL streptomycin and P5Y3 containing 100 µg/mL kanamycin, and kanamycin-sensitive clones were analyzed by colony PCR using primers rsh-FO/rsh-RO.

For complementation studies, fragments containing the *rsh*_US6–1_ ORFs and upstream regions were amplified by PCR using the primer pairs PrshF/PrshR. The PCR products were cloned into the plasmid pCM62 [65] to generate pCM62-rsh via the EcoRI/HindIII restriction site. The plasmid pCM62-rsh was transformed into ∆*rsh* by electroporation (2 kV) using a MicroPulse electroporator (Bio-Rad, Hercules, CA, USA). The complementary strain was verified by qRT-PCR and the tetracycline resistance (9 µg/mL). A Bacterial Genomic DNA Extraction Kit, a Gel Extraction Kit and a Plasmid Miniprep Kit were purchased from TransGen Biotech (Beijing, China) and used according to the manufacturer’s instruction.

### 3.4. Growth Curve

Fresh colonies of US6-1 and its derivatives were first inoculated into P5Y3 broth at the shaking speed of 200 rpm to an OD_600_ value of 1. Then, 250 µL of this seed culture was re-inoculated into 50 mL of fresh P5Y3 broth and incubated statically. The growth curve was drawn from measuring the OD_600_ values. The OD_600_ values were monitored using the Spectrophotometer (Metash, Shanghai, China).

### 3.5. Detection of AHL Accumulation

Strains were tested for AHL accumulation by cross-feeding and extract assays according to the previous study [37]. In the cross-feeding assay, a P5Y3 agar plate was covered with 50 µL X-gal (20 mg/mL stock solution in dimethylformamide). The AHL reporter strain A136 and the tested strains were streaked side by side on the agar plates. Plates were incubated for 24 h, when the activation of the reporter was recorded. A136 versus A136 was used as negative control.

In the extract assay, AHL signals in culture broths were extracted three times by equal volume of ethyl acetate (EA). The EA extracts were combined and evaporated at 55 °C to dry under reduced pressure. The extracts were re-dissolved in 1 mL of EA and passed through a sodium sulfate column to remove water. The extracts were spotted onto LB soft agar plates plus X-gal mixed with A136. The plates were incubated for 12 h at 30 °C and the diameters of the blue stains which represented the quantity of AHL signals in bacterial culture were recorded.

### 3.6. ppGpp Analysis

ppGpp was extracted with formic acid [68]. At 36 h (exponential growth phase) and 72 h (stationary phase) of incubation, bacterial cells were collected by centrifugation and suspended in 1 mL of 0.9% saline. Then 100 µL of 11 M formic acid was added to the suspension. The sample was vigorously mixed and incubated on ice for 30 min. These samples were centrifuged at 10,000× *g* for 10 min at 4 °C. The supernatant was filtered through 0.2 µm filters and stored at −20 °C until use in HPLC analysis.

To assay ppGpp, 100 µL of the extract was subjected to 1100 Series HPLC (Agilent, Santa Clara, CA, USA) by using a ZORBAX SB-C18 column (4.6 × 250 mm, 5 µm, Agilent) at a flow rate of 1.0 mL/min. The mobile phase (pH 6.0) consisted of 125 mM KH_2_PO_4_, 10 mM tetrabutyl ammonium dihydrogen phosphate, 60 mL/L methanol, and 1 g/L KOH [68]. The eluted nucleotides were monitored at 254 nm and identified by comparison with the retention time of 100 µM ppGpp standards (TriLink Biosciences, San Diego, CA, USA). The ppGpp standard was eluted at 71 min under the current conditions.

### 3.7. Identification of AHL Degradation of ∆rsh in the Exponential Growth Phase

To determine whether AHL was degraded by ∆*rsh* in the exponential growth phase, ∆*rsh* was incubated with 2 µM medium-chain AHL (C8-AHL and 3-OH-C8-AHL) for 24 h, 48 h and 72 h, after which the EA extracts were spotted onto AHL reporter plates [69]. The residual AHL was detected by A136. To avoid the influence of the AHL produced by US6-1 in the exponential growth phase, *novI* deletion mutant, which could not produce AHL, was used as positive control. Blank medium with AHL was used as negative control.

### 3.8. RNA Extraction and RNA-seq

Cultures of US6-1, ∆*rsh* and ∆*rsh* (pCM62-rsh) after 24 h, 36 h, 48 h and 72 h of static incubation were frozen in liquid nitrogen and stored at −80 °C. Total RNA extraction was performed with RNAprep pure Cell/Bacteria Kit (Tiangen biotech, Beijing, China) according to the manufacturer’s instructions. RNA concentration and purity were determined at 230 nm using a NanoDrop ND-1000 (Thermo Scientific, Waltham, MA, USA). Genomic DNA contamination was removed and cDNA was synthesized using FastKing gDNA Dispelling RT SuperMix (Tiangen biotech, Beijing, China).

Samples of cDNA of US6-1 and ∆*rsh* at 36 h of incubation (exponential phase) were chosen for RNA-seq analysis. Library construction and sequencing were performed on a BGISEQ-500 platform by Beijing Genomic Institution (Shenzhen, China). All the raw sequencing reads were filtered to remove low quality reads and reads with adaptors, reads in which unknown bases are more than 10%. Clean reads were then obtained. These reads were stored as FASTQ format and then deposited in the NCBI Sequence Read Archive (http://trace.ncbi.nlm.nih.gov/Traces/sra, 22 May 2018) under the accession number SRP148564. HISAT [70] was used to map clean reads to genome of US6-1. For gene expression analysis, the matched reads were calculated and then normalized to reads per kilobaseper million mapped reads using RESM software [71]. The significance of the differential expression of genes was defined according to the combination of the absolute value of the fold change ≥2 and an adjusted *p* value ≤ 0.05 using DESeq2 algorithm [72]. KEGG-based annotation and pathway enrichment analysis was performed using the KEGG pathway database (http://www.genome.jp/kegg/, 25 September 2017). All these KEGG terms were decided by a Fisher’s exact test with phyper function in R language [42]. An adjust *p* value of 0.01 was used as the threshold to obtain significantly over-represented KEGG terms.

### 3.9. Quantitative Real-Time Polymerase Chain Reaction (qRT-PCR)

Quantitative real-time PCR (qRT-PCR) was performed according to protocols described previously [73]. cDNA samples were used as the templates for amplification by using SYBR Premix Ex TaqTM Kit (Tli RNaseH Plus) (TaKaRa, Dalian, China) in Applied Biosystems StepOnePlus^TM^ Real-Time PCR System (Thermo Scientific, Waltham, MA, USA). Primers were designed on the Sangon website (http://www.sangon.com/, 14 January 2018) and listed in Table S4. The following PCR program was used: 95 °C for 30 s, followed by 40 cycles of 95 °C for 5 s, 60 °C for 30 s. A heat dissociation curve (60–95 °C) was checked after the final PCR cycle to determine the specificity of the PCR amplification. The gene expression levels of derivatives of US6-1 relative to the wild-type strain were analyzed using the 2^−∆∆^*^C^*^t^ analysis method [74] (∆*rsh* vs. US6-1 or the complementary strain vs. US6-1). The 16S rRNA gene was used as internal references to normalize cDNA templates. Negative controls with nuclease-free water as templates for each primer set were included in each run. The expression profiles obtained from the RNA-seq were validated by qRT-PCR of 39 representative genes involved in functional groups including AHL production, sigma factor synthesis, RNA degradation, EPS biosynthesis, PAH degradation, sphingolipid production, cell division and shape, GTP synthesis and other functions. The RNA-seq data were considered valid if they met both two requirements. One was that the gene expression (∆*rsh* vs. US6-1) was regulated in the same direction (up or down) in both RNA-seq data and qRT-PCR, and the fold change of genes tested by qRT-PCR was log_2_(x) > 1 or log_2_(1/x) < −1 (i.e., a plain fold change >2) with statistical differences (*p* value < 0.05) [75]. Another was that the gene expression (the complementary strain vs. US6-1) by qRT-PCR was not different significantly or was regulated in the opposite direction in the RNA-seq data. The above valid DEGs were chosen to be clustered. The heat map of their log_2_ expression ratios in the RNA-seq data in strain US6-1 and ∆*rsh* was drawn by the software HemI [43].

### 3.10. Congo Red Binding Assay

The assay to evaluate the EPS production followed the method described previously with minor modification [76]. Cultures were inoculated from a single colony and grown to an OD_600_ of 1 in P5Y3 broth; 10 µL of suspensions were spotted on P5Y3 plates containing 40 µg/mL Congo red (Solarbio, Beijing, China). The plates were grown at 30 °C to assess the morphology of colony biofilm. For quantitative analysis, strains were first cultured in static P5Y3 broth. Then, the bacterial mass was collected at 96 h by centrifuging at 8000 rpm for 5 min and the supernatant was discarded. Determination of biomass was performed gravimetrically as cell wet weight (g). The precipitate was suspended in 1 mL of 100 μg/mL Congo red in 0.9% saline and then incubated for 90 min with shaking at 30 rpm at room temperature. Centrifugation was performed again to separate the mass and solution, and then the OD_490_ of the supernatant was measured. The percentage of Congo red left in the supernatant was calculated by relating to the OD_490_ of 100 μg/mL Congo red in 0.9% saline.

### 3.11. Statistical Analysis

All assays were performed in triplicate and data were expressed as the means and standard deviations (mean ± SD). Analysis of statistical differences was conducted with Student’s *t*-test.

## 4. Conclusions

This is the first transcriptome-wide study of the Rsh regulon in sphingomonads, which is an important step towards understanding the regulatory system of stringent response in sphingomonads. Our study showed that there was a wide range of genes in the Rsh_US6–1_ regulon, including those involved in common traits of sphingomonads such as EPS biosynthesis, PAH degradation and sphingolipid biosynthesis. Furthermore, we focused on the potential molecular mechanisms of Rsh_US6–1_-dependent AHL accumulation. Essential genes for AHL production (*novI* and *novR*) were positively regulated by Rsh_US6–1_ in the exponential growth phase. Several factors including the ECF sigma factor, DksA, ATP-dependent Lon protease and RNA degradation enzymes might be involved in the ppGpp-dependent expression of *novI* and *novR*. Future studies will focus on the validation of the above proposed mechanisms, which might provide an insight into the complicated cross-talk between stringent response and QS in sphingomonads.

## Figures and Tables

**Table 1:** Relative expression levels of *novI* and *novR* by quantitative reverse transcription polymerase chain reaction (qRT-PCR). RNA samples were extracted from bacterial cultures after 24 h, 36 h, 48 h and 72 h. The levels of gene expression in US6-1, ∆*rsh, ∆rsh* (pCM62-rsh) were normalized to 16S rRNA gene, and the relative levels in ∆*rsh* and *∆rsh* (pCM62-rsh) to that in the wild-type strain (set as value of 1) were reported. Values shown are the average of biological triplicate experiments with standard deviations (mean ± standard deviation (SD)). “*” represents *p* < 0.05; “**” represents *p* < 0.01; “***” represents *p* < 0.001.

Genes	Strains	24 h	36 h	48 h	96 h
*novI*	US6-1	1.166 ± 0.773	1.060 ± 0.466	1.115 ± 0.647	1.039 ± 0.356
	∆*rsh*	0.261 ± 0.083	0.011 ± 0.002 *	0.020 ± 0.004 *	0.026 ± 0.004 **
	∆*rsh* (pCM62-rsh)	0.501 ± 0.085 ***	0.648 ± 0.216	0.753 ± 0.097	3.975 ± 1.547 *
*novR*	US6-1	1.012 ± 0.185	1.006 ± 0.129	1.001 ± 0.055	1.029 ± 0.314
	∆*rsh*	0.119 ± 0.018 **	0.068 ± 0.005 ***	0.547 ± 0.017 ***	0.881 ± 0.354
	∆*rsh* (pCM62-rsh)	0.601 ± 0.050 **	0.463 ± 0.076	0.543 ± 0.054	2.486 ± 0.972

**Table 2:** Bacterial strains and plasmids used in this study.

Strains or Plasmids	Relevant Traits	Source or Reference
Strains		
*N. pentaromativorans*		
US6-1	Wild type (JCM 12182)	Microbe division JCM
∆*rsh*	US6-1, *rsh*_US6-1_ deletion mutant, Rif^r^	This study
∆*rsh* (pCM62-rsh)	∆*rsh* with the plasmid pCM62-rsh, Tc^r^	This study
∆*novI*	US6-1, *novI* deletion mutant, Rif^r^	Lab stock
*E. coli*		
DH5α	F- *hsdR17 endA1 thi-1 gyrA96 relA1 supE44∆lacU169* (ψ80*dlacZ*∆M15)	TransGen Biotech
S17-1 (λpir)	*E. coli* K-12 Tp^r^ Sm^r^ *recA thi hsdRM*^+^ RP4::2-Tc::Mu::Km Tn7, *λpir* phage lysogen	[62]
*A. tumefaciens* A136 (pCF218) (pCF372)	*traI*-*lacZ* fusion; AHL biosensor; Tc^r^ Sp^r^	[63]
Plasmids		
pMD19-T	T-vector, Amp^r^	TaKaRa, Dalian, China
pAK405	Plasmid for allelic exchange and markerless gene deletions; Km^r^	[64]
pAK405-rsh	pAK405 with fusion of up- and downstream regions of *rsh*_US6-1_; Km^r^	This study
pCM62	Broad-host-range cloning vector; IncP origin of replication; Tc^r^	[65]
pCM62-rsh	pCM62 with *rsh*_US6-1_ ORF and 502 bp of upstream region; Tc^r^	This study

## Supplementary Materials

The following are available online at http://www.mdpi.com/1422-0067/19/9/2631/s1, Table S1: Results of mapping clean reads of RNA-Seq to the reference genome of US6-1; Table S2: Complete list of DEGs according to RNA-seq (∆*rsh* vs. US6-1); Table S3: Validation of the RNA-seq data by qRT-PCR of 39 representative genes; Table S4: Primers used in this study.

## Abbreviations

AHL	*N*-acyl-l-homoserine Lactone
QS	Quorum Sensing
DEGs	Differentially Expressed Genes
PAH	Polycyclic Aromatic Hydrocarbon
ECF	Extracytoplasmic Function
SPT	Serine Palmitoyltransferase
EPS	Exopolysaccharide
